# Betel-quid Use Is Associated with Depression

**DOI:** 10.7150/ijms.119520

**Published:** 2026-01-01

**Authors:** Perl Han Lee, Jiun-Hung Geng, Szu-Chia Chen, Chien-Hung Lee, Chih-Hung Ko

**Affiliations:** 1 Graduate Institute of Medicine, College of Medicine, Kaohsiung Medical University, Kaohsiung 80708, Taiwan.; 2 Graduate Institute of Clinical Medicine, College of Medicine, Kaohsiung Medical University, Kaohsiung 807378, Taiwan.; 3 Faculty of Medicine, College of Medicine, Kaohsiung Medical University, Kaohsiung 807378, Taiwan.; 4 Research Center for Environmental Medicine, Kaohsiung Medical University, Kaohsiung 807378, Taiwan.; 5 Department of Public Health and Environmental Medicine Research Center, Kaohsiung Medical University, Kaohsiung 80708, Taiwan.; 6 Department of Psychiatry, Kaohsiung Medical University Hospital, Kaohsiung 80708, Taiwan.; 7 Department of Psychiatry, Kaohsiung Municipal Siaogang Hospital, Kaohsiung, Taiwan.; 8 Department of Urology, Kaohsiung Municipal Siaogang Hospital, Kaohsiung 812015, Taiwan.; 9 Department of Urology, Kaohsiung Medical University Hospital, Kaohsiung Medical University, Kaohsiung 807377, Taiwan.; 10 Department of Urology, School of Medicine, College of Medicine, Kaohsiung Medical University 807378, Kaohsiung, Taiwan.; 11 Department of Internal Medicine, Kaohsiung Municipal Siaogang Hospital, Kaohsiung Medical University 812015, Kaohsiung, Taiwan.; 12 Division of Nephrology, Department of Internal Medicine, Kaohsiung Medical University Hospital, Kaohsiung Medical University, Kaohsiung 807377, Taiwan.

**Keywords:** Betel-quid use, depression, risk factors, epidemiologic study

## Abstract

**Background:** Betel-quid use is linked to cancer and other adverse health effects. However, its potential impact on depression has not been well studied.

**Objective:** To investigate the association between betel-quid use and depression.

**Methods:** We analyzed the data of 43,636 men from the Taiwan Biobank. Participants were divided into two groups on the basis of betel-quid use: never-user and ever-user groups. Depression was defined according to self-reported medical history in the questionnaire. Logistic regression was used to assess the association between betel-quid use and depression.

**Results:** The average age of the participants was 49.89 ± 11.36 years. Among them, 16.31% were ever-users of betel quids. In the never-user group (n = 36,521), 828 participants (2.27%) reported a history of depression. However, 75 of 2,592 participants in the ever-user group (2.89%) reported a similar history. The adjusted odds ratio for depression in the ever-user group compared with the never-user group was 1.265, indicating its association with depression. Daily consumption of 11-30 betel quids was associated with depression compared with consumption of ≤10 betel quids. However, no association was found for those consuming more than 30 betel quids daily.

**Conclusions:** Betel-quid use is associated with depression. The association between betel-quid use and depression may not be linear, suggesting a complex dose-response effect.

## Introduction

Betel quids constitute a preparation that typically consists of fresh, unripe, or dried areca catechu nut that is often wrapped in a piper leaf and occasionally combined with tobacco and smeared with aqueous lime 1. Areca nut which is a key ingredient of the betel quid is the 4th most commonly abused substance 2. Betel quids and areca nuts are linked to an increase risk of several cancers, including those of the oral cavity 3, larynx, esophagus 4, lung, breast 5, liver, pancreas 6, and stomach 7, and are classified as Group 1 human carcinogens by the International Agency for Research on Cancer 8. In addition to its carcinogenic effects, betel-quid use is associated with psychiatric disorders.

Areca-nut psychosis, though rare, was first reported in 1966 9. It is characterized by auditory hallucinations and delusions, typically occurring in long-term betel-quid users who have recently increased their consumption 9. A recent case report described a 30-year-old man with no prior medical history who developed psychosis with alarming behaviors after an increase in betel-quid intake 10. Conversely, betel quids have been found to interact with muscarinic receptors and can alleviate both positive and negative symptoms of schizophrenia 11. 12 identified an association between betel-quid use and common mental disorders, including anxiety, depression, sleep disturbance, and somatic symptoms, as assessed by the 12-item Chinese Health Questionnaire (CHQ-12). Additionally, a study reported that a diagnosis of bipolar disorder and current tobacco use were predictors of betel-quid use 13.

Arecoline is among the most extensively studied alkaloids in areca nut owing to its effects on depression in animal models. One study reported that arecoline alleviated depression-like behaviors in adult mice, as determined from the forced-swim test and tail-suspension test 14; the study analyzed anterior cingulate cortex (ACC) brain slices and revealed that muscarinic receptors were the primary target of arecoline in the ACC. Another study conducted microarray and quantitative reverse transcription polymerase chain reaction and demonstrated that arecoline and areca nut extract inhibited monoamine oxidase A (MAO-A) activity in rats 15. 16 also used liquid chromatography-tandem mass spectrometry and Western blotting; their results demonstrated that areca nut alleviated stress-induced changes in 5-hydroxytryptamine (5-HT) and dopamine levels and increased protein levels of brain-derived neurotrophic factor (BDNF), TrkB, PI3K, and other factors in brain tissues 16. Moreover, 17 reported that dichloromethane exhibited antidepressant activity through MAO-A inhibition, although this effect was not observed with the major alkaloids. MAO-A activity in human plasma was also observed to increase with cumulative use of betel quids 15. These findings indicate that the antidepressant effect of areca nut may be associated with BDNF signaling and elevated levels of monoamines (such as serotonin and dopamine) due to MAO-A inhibition.

18 analyzed areca nut samples by using ultrahigh-performance liquid chromatography-tandem mass spectrometry, and they identified a total of 873 metabolites. Through network pharmacology analysis, they predicted that certain amino acids and their derivatives (e.g., L-phenylalanine, L-tyrosine) and phenols could be associated with depression. This prediction is supported by previous research showing reduced levels of phenylalanine and tyrosine in patients with depression 19. Because tyrosine is a precursor for dopamine synthesis and because decreased dopamine levels are linked to depression, one can reasonably speculate that these metabolites may play a major role in depression interventions 19. Furthermore, the main depression-related genes targeted by these metabolites, such as CES2 and ARG1, are involved in various inflammatory response pathways, including the IFN-gamma pathway, TNF receptor signaling pathway, and p38 MAPK signaling pathway. This suggest that areca nut may be effective in treating depression through its anti-inflammatory properties 18, which is consistent with the inflammatory hypothesis of depression 20, 21. Additionally, a recent study highlighted the role of chemokines in depression treatment after examining the action and mechanism of Areca Thirteen Pill, a traditional Mongolian medicine 22. This study revealed that Areca Thirteen Pill upregulates the AC-cAMP-PKA signaling pathways in the hippocampus and prefrontal cortex of a depression rat model 23.

Although animal studies can provide basic insights into the biological and physiological processes of areca nut, real-world human medical research is essential for understanding the effects of areca nut on the human mind and body. Only a few human studies have explored the association between betel-quid use and depression, and many of these studies were conducted in Taiwan. A cross-sectional study of more than 9,000 adolescents aged 12 to 19 years reported higher rates of betel-quid, tobacco, alcohol, sedative, and illicit drug use among those with severe depressive symptoms compared with nondepressive individuals; those with minor depressive symptoms also had higher rates of substances use, except for illicit drugs 24. The Taiwan Longitudinal Study on Aging conducted in 1989 and targeting elderly individuals reported that past tobacco use and current betel-quid use were associated with an increased risk of new-onset depressive symptoms 25. Conversely, another study involving approximately 2,000 participants indicated that neither tobacco nor betel-quid use predicted becoming a healthy octogenarian 26. Another study analyzing the data of 1,331 participants from the 2005 Taiwan National Health Interview Survey revealed a significant association between betel-quid use and a decreased risk of depression, as determined from a multivariate logistic regression analysis 27. 28 included approximately 500 Taiwanese participants and observed that betel-quid users had significantly lower scores on extraversion and higher scores on fatigue, anger, tension, and depression, suggesting an association between betel-quid use and low extraversion and negative mood. In addition to this Taiwanese research, a study conducted in the Federated States of Micronesia assessed areca-nut use, anxiety, and depression among seventh- and eighth-grade students; the study indicated no association between areca-nut use and depression or anxiety, but the study included a small sample, consisting of only 100 participants 29.

According to our review of the literature, no study has explored the dose-dependent effect of betel-quid use on depression. One clinical study involving 204 participants reported that patients with depression had a higher prevalence of betel-quid use 30. The study followed 20 depressed patients from the betel-quid use group for two consecutive years and observed a significant reduction in both the amount and frequency of betel-quid consumption after antidepressant treatment. Daily consumption decreased from 44.5 to 5.3 betel-quid pieces, and the frequency decreased from 5.7 to 0.9 days per week. Although this study did not directly address a dose-dependent effect, it suggested a potential reverse relationship between betel-quid use and depression risk. Accordingly, to address these research gaps, the objectives of the present study were to conduct a large-scale investigation into the association between betel-quid use and depression and to explore the dose-dependent effect of betel-quid use on depression.

## Materials and methods

### Taiwan biobank and study design

The Taiwan Biobank, established by the Ministry of Health and Welfare in Taiwan, is a comprehensive human biological database comprising a cohort of more than 100,000 Taiwanese individuals aged 30 to 70 years who do not have a cancer diagnosis. It collects data on biological markers, environmental factors, and lifestyle habits to support research aimed at identifying strategies to address the onset, progression, and treatment of diseases and to enhance public health 31-33.

To investigate the association between betel-quid use and depression among men, a cross-sectional study was conducted using data from the Taiwan Biobank collected between 2008 and 2019. All participants provided written informed consent during enrollment. Initially, 43,848 men were included in this study; however, 212 men were excluded due to incomplete data, resulting in a final sample of 43,636 men. The study was approved by the Institutional Review Board of Kaohsiung Medical University Hospital (KMUHIRB-E(I)-20190398).

### Betel-quid use assessments

The participants were asked the following questions: “Have you ever chewed betel quids?” Participants who responded “never” or “only once or twice” were assigned to the never-use group, and those who responded “yes” were assigned to the ever-use group. For those in the ever-use group, the following additional questions were asked: “When did you start chewing betel quids?”; “Are you currently a betel-quid user?”; and “How many betel-quid pieces do you consume each day?” On the basis of their responses, the participants were classified into four categories: “never-use,” “≤ 10 betel-quid pieces per day,” “11 to 30 betel-quid pieces per day,” and “> 30 betel-quid pieces per day.”

### Self-reported diagnosed depression

Depression was defined on the basis of a self-reported history of diagnosed depression by using a simple “yes”/ “no” question: “Have you ever been diagnosed with depression?” If the participants answered “yes,” they were further asked, “When were you diagnosed with depression?”

### Statistical analysis

Data were analyzed using the SPSS (Statistical Package for Social Sciences, version 20.0.0) package. A *p* value of <.05 was considered statistically significant for all analyses. Categorical variables are presented as counts and percentages, and continuous variables are expressed as means ± standard deviations. Chi-square tests were conducted to examine differences among categorical variables, and independent t-tests were used to assess differences among continuous variables. Logistic regression analysis was performed to evaluate the association between betel-quid use and self-reported diagnosed depression. To investigate the dosage-dependent effect of betel-quid use on depression, a subgroup of 39,113 participants with complete information was analyzed. These participants were divided into four groups, as described earlier, and logistic regression was applied to analyze the association between the frequency of betel-quid use and depression within these subgroups.

## Results

The demographic data and characteristics of the 43,636 men are presented in Table 1. Of the participants, 36,521 (83.69%) were in the never-use group, and 7,115 (16.31%) were in the ever-use group. Compared with those in the never-use group, the participants in the ever-use group were generally older, had a higher body mass index, engaged in less physical activity, and had lower educational levels. Additionally, the ever-use group exhibited a significantly higher prevalence of hypertension, dyslipidemia, diabetes mellitus, alcohol use, and tobacco use.

A univariate binary logistic analysis was performed to examine the association between each parameter and depression, and the results are presented in Table 2. Older age, hypertension, dyslipidemia, diabetes mellitus, tobacco use, and betel-quid use were significantly associated with higher odds of depression. Specifically, the participants in the ever-use group had a 1.32-fold increase in the odds of depression compared with those in the never-use group [odds ratio, 1.324; 95% confidence interval (CI), 1.136-1.543]. Conversely, being married was associated with lower odds of depression.

To adjust for cohort effects, parameters that were significant in the univariate analysis (with a *p* value of < .05) were included in a multivariate binary logistic regression analysis, and the results are listed in Table 3. Older age, dyslipidemia, tobacco use, and betel-quid use were significantly associated with higher odds of depression. Specifically, the participants in the ever-use group had a 1.27-fold increase in the odds of depression compared with those in the never-use group (adjusted odds ratio, 1.265; 95% CI, 1.072-1.493).

Table 4 presents the results of the association between daily betel-quid use amounts and depression. Participants using 11 to 30 betel-quid pieces per day had a significant 1.43-fold increase in the odds of depression compared with those in the never-use group (adjusted odds ratio, 1.429; 95% CI, 1.034-1.974). However, the adjusted odds ratio for participants using more than 30 betel-quid pieces per day did not show a significant difference.

## Discussion

In Taiwan, betel-quid stands and roadside booths are very common, often featuring neon advertising boards and scantily dressed women to promote their businesses 34. Betel-quid chewing is socially acceptable across all age group 35, and it is particularly popular among working-class men, who use it for increased energy and enhanced occupational performance 36. Previous studies have reported that up to 92.6% of betel-quid users are also smokers 37, and the act of sharing betel quids and cigarettes with coworkers and clients can foster relationships and facilitate business interactions. Men in this group are often considered to have lower educational levels and social status. Our study findings are consistent with this observation; specifically, we determined that betel-quid users tended to have lower educational levels and to have higher percentages of hypertension, dyslipidemia, and diabetes mellitus, likely due to polysubstance use.

Several studies have demonstrated an association between depression and metabolic syndrome. A meta-analysis that included nine studies with more than 22,000 participants concluded that depression could be an independent risk factor for hypertension 38. Patients with type 2 diabetes were reported to be at a relatively high risk of lifetime major depressive disorder 39. Moreover, diabetes was noted to increase the risk of depression, and depressive symptoms, in turn, elevated the risk of diabetes mellitus 40. Another meta-analysis reported that both high and low levels of serum LDL-C were related to increased odds of depression, suggesting that dyslipidemia is also associated with depression 41. This is consistent with our findings, which demonstrate that participants with hypertension, dyslipidemia, and diabetes mellitus had significantly higher odds of depression. Considering the various factors associated with depression, we conducted a multivariate binary logistic analysis to control for potential confounding effects. Our results indicate that betel-quid use was still significantly associated with depression after adjustment for these confounders.

Substance use is common among patients with mood disorders. Substance use and mood disorders may share a common pathophysiological factor, including genetic or environmental risk factors 42. Substances can induce mood disorders through their effects on neurotransmitter systems, either due to use, abuse, or withdrawal 43. Additionally, substances may be used as a form of self-medication to alleviate mood symptoms 44. Previous studies have reported a higher prevalence of betel-quid use among patients with depression, and those who used betel quids tended to exhibit more depressive symptoms 24, 30. A longitudinal study observed that betel-quid use was associated with an increased risk of new-onset depressive symptoms 25, whereas another study reported a significant association between betel-quid use and a decreased risk of depression 27. Such conflicting results are common in the literature. Many previous studies were limited by small sample sizes, ranging from as few as 100 to approximately 9,000 participants. A strength of our study is its large sample size of over 40,000 participants, which enabled us to provide robust evidence for the association between betel-quid use and depression.

Although several studies reported a significant association between betel-quid use and depression, the mechanism underlying this association remains unclear. One possible mechanism is related to betel-quid's potential antidepressant activity. Areca nuts have a long history of use in traditional Chinese medicine for treating depression, particularly in Mongolian regions 45. Several studies have demonstrated that areca nut extract could target 141 depression-related genes 18 and inhibit monoamine oxidase A, leading to increased levels of serotonin and norepinephrine 17, 46. Betel quids may have been used by patients as a form of self-medication to alleviate symptoms of depression. However, despite its antidepressant activity, self-medication may not fully explain the increased betel-quid use observed in patients with depression. A previous study reported that patients with depression who used betel quids had more severe depressive symptoms compared with those who did not use them, particularly if they had poorly responded to antidepressant treatment 30. This discrepancy might be explained by the dysregulation of MAO-A activity. Betel-quid use has been reported to increase catecholamine levels, which can lead to elevated MAO-A activity, potentially exacerbating depressive episodes 15. Dysregulation and overactivity of MAO-A in the brain could be associated with greater severity of depressive episodes 47. This suggests that the association between betel-quid use and depression is complex.

Another possible mechanism is related to betel-quid use withdrawal. Betel-quid use affects both central and autonomic nervous systems function 48, and withdrawal symptoms such as reduced energy, reduced concentration, appetite changes, sleep disturbances, anxiety, and irritability may occur 1 to 2 days after cessation 49. Antidepressant treatment was previously reported to reduce both the amount and the frequency of betel-quid use 50, and the addictiveness of betel-quid use was significantly reduced following antidepressant therapy 30. These findings suggest that continuous betel-quid in patients with depression might be an attempt to prevent the development of withdrawal symptoms.

Our study revealed that participants consuming 11-30 betel quids use per day had an association with depression compared with those with lower frequencies of use (never or ≤10 betel quids per day). This supports the hypothesis that increased frequency of betel-quid consumption correlates with depression. However, no association was found for participants using > 30 betel quids per day. We could not determine whether this result was due to a complex association mechanism or limitations in sample size. For example, a previous study reported a decreased risk of depression with betel-quid use 27.

An animal study that administered 100 mg/kg/day of areca nut extract to rats observed reductions in MAO-A mRNA expression in brain cells on days 15, 30, and 45, with the corresponding fold changes being 0.79, 0.20, and 0.25, respectively 15. This suggests that cumulative betel-quid use might affect MAO-A activity in the human brain. A similar study could be designed with groups receiving different amounts of areca nut extract, followed by examination of MAO-A mRNA expression. This animal model could provide a valuable first step in understanding the dose-response effect of betel quids.

Our study is the first large-scale investigation with over 43,000 participants to examine the association between betel-quid use and depression. Although our findings provide strong support for this association, our study has several limitations. First, reviewing each medical record or conducting diagnostic interviews for all participants was challenging, making reliance on self-reported diagnosed depression a notable limitation. Hence, incorporating measures of symptom severity for depression would enhance the study, particularly for analyzing the dose-response effect between betel-quid use and depression risk. Second, cultural background and lifestyle factors vary across different countries. Despite the large sample size, all subjects in this study were from Taiwan, which may limit the generalizability of our findings. Future studies with inclusion of participants from different countries are needed to address this limitation.

In conclusion, our study indicated that betel-quid use is associated with depression. The dose-response association between betel-quid use and depression may not follow a simple linear trend.

## Author contributions

PHL, JHG, and SCC designed the study, carried out the data analysis, and drafted the initial manuscript. CHK, and CHL contributed with writing, commenting, and critically reviewing the manuscript. All authors have read and approved the final manuscript.

## Ethics approval and consent to participate

The study was conducted according to the guidelines of the Declaration of Helsinki and approved by the Institutional Review Board of Kaohsiung Medical University Hospital (KMUHIRB-E(I)-20210058).

## Availability of data and materials

The datasets generated and/or analyzed during the current study are not publicly available due to reasons of sensitivity but are available from the corresponding author on reasonable request.

## Figures and Tables

**Table 1:** Clinical characteristics of the study participants classified by betel-quid use.

		Betel-quid Use		
Characteristics	Total (N = 43,636)	Never (N = 36,521)	Ever (N = 7,115)	*p* Value
Age, years	49.89 ± 11.36	49.75 ± 11.61	50.58 ± 9.98	<0.001***
BMI, kg/m^2^	25.37 ± 3.56	25.21 ± 3.49	26.14 ± 3.79	<0.001***
Physical activity ^a^, n	18,490 (42.37%)	15,906 (43.55%)	2,584 (36.32%)	<0.001***
Married, n	37,735 (86.48%)	31,271 (85.62%)	6,464 (90.85%)	<0.001***
Education level, n				<0.001***
≤ Elementary school	1,312 (3.01%)	882 (2.42%)	430 (6.04%)	
Middle-to-high school	13,202 (30.25%)	9,213 (25.23%)	3,989 (56.06%)	
≥ College	29,122 (66.74%)	26,426 (72.36%)	2,696 (37.89%)	
Hypertension, n	7,333 (16.80%)	5,817 (15.93%)	1,516 (21.31%)	<0.001***
Dyslipidemia, n	4,053 (9.29%)	3,168 (8.67%)	885 (12.44%)	<0.001***
Diabetes mellitus, n	2,960 (6.78%)	2,312 (6.33%)	648 (9.11%)	<0.001***
Alcohol use ^b^, n	8,156 (18.69%)	5,201 (14.24%)	3,135 (44.06%)	<0.001***
Tobacco use^ c^, n	8,167 (18.72%)	5,198 (14.23%)	2,969 (41.73%)	<0.001***

Abbreviations: BMI: body mass index; ^a^ defined as at least 3 times a week with 30 minutes each; ^b^ defined as at least 150cc a week for 6 months; ^c^ defined as at least 6 months and still ongoing; *** significance at p < 0.001.

**Table 2:** Association between parameters and depression in univariable binary logistic analysis (N=43,636).

Parameters	Odds Ratio	95% CI	*p*
Age (per 1 year)	1.015	1.009-1.020	< 0.001***
BMI (per 1 unit)	0.999	0.982-1.017	0.932
Physical activity^ a^ (yes vs. no)	1.076	0.951-1.218	0.245
Married (yes vs. no)	0.577	0.496-0.672	< 0.001***
Education level (≤ elementary school vs. others)	0.884	0.792-0.987	0.028*
Hypertension (yes vs. no)	1.477	1.275-1.711	< 0.001***
Dyslipidemia (yes vs. no)	2.093	1.776-2.466	< 0.001***
Diabetes mellitus (yes vs. no)	1.580	1.288-1.939	< 0.001***
Alcohol use ^b^ (yes vs. no)	1.158	0.995-1.347	0.058
Tobacco use ^c^ (yes vs. no)	1.205	1.037-1.399	0.015*
Betel-quid use (ever vs. never)	1.324	1.136-1.543	< 0.001***

Abbreviations: BMI: body mass index and CI: confidence interval; ^a^ defined as at least 3 times a week with 30 minutes each; ^b^ defined as at least 150cc a week for 6 months; ^c^ defined as at least 6 months and still ongoing; * significance at p < 0.05; ** significance at p < 0.01; *** significance at p < 0.001.

**Table 3:** Association between parameters and depression in multivariate binary logistic analysis (N=43,636).

Parameters	Adjusted Odds Ratio	95% CI	*p*
Age (per 1 year)	1.025	1.018-1.032	< 0.001***
Married (yes vs. no)	0.375	0.314-0.448	< 0.001***
Education level (≤ elementary school vs. others)	1.016	0.902-1.144	0.793
Hypertension (yes vs. no)	1.135	0.964-1.335	0.128
Dyslipidemia (yes vs. no)	1.826	1.527-2.183	< 0.001***
Diabetes mellitus (yes vs. no)	1.122	0.902-1.395	0.300
Tobacco use^ a^ (yes vs. no)	1.193	1.019-1.396	0.028*
Betel-quid use (ever vs. never)	1.265	1.072-1.493	0.005**

Abbreviations: BMI: body mass index and CI: confidence interval; ^a^ defined as at least 6 months and still ongoing; * significance at p < 0.05; ** significance at p < 0.01; *** significance at p < 0.001.

**Table 4:** Association between frequency of betel-quid use and depression (N=39,113)

Betel-quid Use Frequency	No. of Cases	Number at Risk	Adjusted Odds Ratio	95% CI	*p*
Never use	828	36,521	1.000	-	-
≤ 10 betel quids per day	22	974	0.952	0.618-1.466	0.822
11-30 betel quids per day	43	1,242	1.429	1.034-1.974	0.031*
> 30 betel quids per day	10	376	0.949	0.499-1.804	0.874

Abbreviations: CI: confidence interval; * significance at p < 0.05.
